# Supplementary material for: From sequence to enzyme mechanism using multi-label machine learning
Source: BMC Bioinformatics. 2014 May 19;15:150. doi: 10.1186/1471-2105-15-150 (PMC4229970; doi:10.1186/1471-2105-15-150)
Supplement: Additional file 2 — Java code of ml2db. Additional file ml2db_code.tar.gz contains the Java source code to run the multi-label machine learning experiments and save the results to database. The code’s Javadoc is included. [file 1471-2105-15-150-S2.zip › additional file 2/ml2db/utils/doc/uk/ac/standrews/utils/main/package-summary.html]

uk.ac.standrews.utils.main


JavaScript is disabled on your browser.


- Overview
- Package
- Class
- Use
- Tree
- Deprecated
- Index
- Help

- Prev Package
- Next Package

- Frames
- No Frames

- All Classes

# Package uk.ac.standrews.utils.main

- Interface Summary

  | Interface | Description |
  | --- | --- |
  |  |  |
  | --- | --- |
  | Initialisable | A class having a boolean variable telling whether the initialisation has completed correctly |
- Class Summary

  | Class | Description |
  | --- | --- |
  |  |  |
  | --- | --- |
  | BooleanArrayPool | Class |
  | EntrezUtils | Utilities for Entrez services. |
  | FileUtils | Utils to read, write files etc. |
  | Initialised |  |
  | LoggerCreator |  |
  | LogUtils | Utilities to log error messages |
  | NumberUtils | Class |
  | PathUtils | Paths to utilities folders and files |
  | PropertiesUtils | Utilities to manipulate Java properties and properties text files |
  | ReflectionUtils | Class |
  | RegExpUtils |  |
  | Scripts | Class |
  | SystemUtils | Utilities for the System class. |
  | TimeUtils | Utilities to manipulate time objects, create timestamps etc. |
  | Utils | Various utilities to manipulate vectors of numbers, get a timestamp etc. |

- Overview
- Package
- Class
- Use
- Tree
- Deprecated
- Index
- Help

- Prev Package
- Next Package

- Frames
- No Frames

- All Classes
